# Impact of *ABCB1* Gene Polymorphisms and Smoking on the Susceptibility Risk of Chronic Myeloid Leukemia and Cytogenetic Response

**DOI:** 10.29252/ibj.25.1.54

**Published:** 2020-08-25

**Authors:** Fatemeh Mohammadi, Mohammad Shafiei, Dlnya Assad, Golale Rostami, Mohammad Hamid, Ali Mohammad Foroughmand

**Affiliations:** 1 Department of Biology, School of Science, Shahid Chamran University of Ahvaz, Ahvaz, Iran;; 2 Biotechnology and Biological Science Research Center, Shahid Chamran University of Ahvaz, Ahvaz, Iran;; 3 Department of Biology, College of Science, Sulaimani University, Sulaymanyah, Iraq;; 4 Department of Molecular Medicine, Biotechnology Research Center, Pasteur Institute of Iran, Tehran, Iran

**Keywords:** ATP binding cassette transporter subfamily B, Imatinib mesylate, Smoking

## Abstract

**Background:**

IM, a strong and selective TKI, has been approved as the front line of treatment in CML patients. In spite of satisfactory results of imatinib in the treatment of patients with CML, patients with treatment failure or suboptimal response developed resistance that might be because of pharmacogenetic variants. This study attempted to evaluate the influence of *ABCB1* gene polymorphisms and smoking on CML risk and resistance to imatinib.

**Methods:**

*ABCB1* (c.1236C>T, c.3435C>T) polymorphisms were genotyped in 98 CML patients and 100 sex- and age-matched healthy subjects by PCR-RFLP method, followed by sequencing. The patients were evaluated for cytogenetic response by the standard chromosome banding analysis in regular intervals.

**Results:**

Our results showed that c.1236CC genotype was significantly associated with imatinib resistance (OR = 3.94; *p *= 0.038). Analysis of the joint of SNP-smoking combination showed that smokers with c.1236TT/CT and c.1236CC genotypes had the increased risk of CML (OR = 6.04; *p *= 0.00 and OR = 4.95, *p *= 0.005) and treatment failure (OR = 5.36, *p *= 0.001 and OR = 15.7, *p *= 0.002), respectively. Smokers with c.3435TT/CT and c.3435CC genotypes also displayed the elevated risk of CML development (OR = 6.01, *p *= 0 and OR = 4.36, *p *= 0.011) and IM resistance (OR = 5.61,* p *= 0.001 and OR = 13.58, *p *= 0.002), respectively.

**Conclusion:**

Our findings suggest that c.1236CC genotype has clinical importance in the prediction of treatment outcome with IM, and smoking could have a synergistic role in CML risk and IM resistance.

## INTRODUCTION

Chronic myeloid leukemia is a myelo-proliferative disorder resulting from reciprocal chromosome translocation t(9; 22)(q34; q11) or the *BCR-ABL1* fusion gene^[^^1^^]^. Imatinib is a strong TKI and prescribed as the front line of treatment in CML patients. IM inhibits the autophosphorylation of *BCR-ABL1* oncoprotein in CML patients^[^^2^^]^. Despite satisfactory results of imatinib in the treatment of CML patients, there are still patients with treatment failure or suboptimal response^[^^3^^]^. 

Two groups of mechanisms have been proposed for resistance to IM that include BCR-ABL1-dependent and -independent mechanisms. *BCR-ABL1*-dependent mechanism includes point mutations in the tyrosine kinase domain and amplification of *BCR-ABL1* gene. The point mutations account for about 50% of patients who do not achieve favorable response^[^^4^^,^^5^^]^. Pharmacogenetic variability is a *BCR-ABL1*-independent mechanism, which impacts the pharmacokinetics of IM and might be a resistance-causing mechanism^[^^5^^]^. Studies have reported that *ABCB1/MDR1* gene SNPs may modulate the incidence of cancer rate^[^^6^^,^^7^^]^ or induce resistance to IM^[^^5^^,^^8^^]^. *ABCB1* gene encodes P-gp, which is located on the long arm of chromosome 7 (7q21.12). 

To date, over 50 SNPs within *ABCB1* gene have been recognized. Two polymorphisms have the highest biological and clinical importance: the first is a nucleotide change at the position 1236 in exon 12 (c.1236C>T, rs1128503) and the second is located at the position 3435 in exon 26 (c.3435C>T, rs1045642)^[^^9^^]^. P-gp is a transporter protein that controls the regulation of the efflux of endogenous and xenobiotics compounds between cells and their environment. It acts as an efflux pump and protects cells against materials such as organic cations, toxins, and antibiotics^[^^10^^,^^11^^]^. Numerous common coding variants in *ABCB1* have been studied for their potential influence on P-gp expression, function, and disease risk^[^^10^^]^. The P-gp may contribute to mutagenesis via the cumulation of exogenous toxins in cells and increase the risk of cancer^[^^11^^,^^12^^]^. Some investigations have reported that the overexpression of P-gp enhances the clearance of the drug, resulting in decrease drug availability in the cells and confer resistance to IM^[^^8^^,^^13^^]^. IM is a substrate for *ABCB1* transporter, and *ABCB1* polymorphisms may influence the pharmacokinetic and intracellular concentration of IM. This transporter plays a role in creating resistance by deporting IM from hematopoietic cells^[^^5^^]^. 

Pharmacogenetic studies contribute to the evaluation and prediction of IM response in CML patients. Therefore, we aimed to determine the effect of *ABCB1* polymorphisms, their combinations together, and the joint effect of SNP-smoking combination on CML risk and resistance to imatinib in Iranian CML patients.

## MATERIALS AND METHODS

**Study subjects**

The study population was composed of 98 patients who were diagnosed with CML at Arad hospital (Tehran, Iran, n = 88) and Saba Oncology Clinic (Isfahan, Iran, n = 10). All patients were on imatinib therapy (300-800 mg/day), and the median duration of IM treatment was 46 months, ranging from 10 to 175 months. Moreover, 100 age-, sex- and ethnicity (Fars)-matched healthy individuals without the history of cancer and other chronic diseases were selected. The cytogenetic responses to imatinib were categorized based on the European Leukemia Net recommendation^[^^14^^]^, and the absence of any philadelphia chromosome positivity (Ph^+^) cells was considered a CCyR. The patients were scored for 12 months as responders (CCyR, Ph^+^ = 0%, n = 44) or non-responder (non-CCyR, Ph^+^ > 0%, n = 54). Smoking habits were categorized as active (subjects who have smoked ≥ 1 package of cigarettes daily), passive (those who were exposed to smoke or have smoked < 1 package cigarettes daily), and never (non)-smokers. 

**Clinical evaluation**

Assessment of clinical response was performed based on European Leukemia Net criteria^[^^14^^,^^15^^]^. The cytogenetic response was evaluated at six-month intervals based on the standard protocol for chromosomal banding of bone marrow cell metaphases as previously described^[^^16^^]^. CCyR was defined as 0% Ph^+^ chromosome in minimum 20 metaphases.

**Molecular analysis**

DNA was extracted from the blood of the subjects under study using the salting-out method^[^^17^^]^. PCR-RFLP method was used for the amplification of the SNPs *ABCB1* (1236C>T, 3435C>T). PCR products were digested by suitable enzymes according to the manufacturer's instructions (Thermo Fisher Scientific, USA). For checking the result of digestion, 10% of samples were sequenced. Primer sequences, restriction enzymes, and PCR conditions are shown in Table 1. 

**Statistical analysis**

Hardy-Weinberg equilibrium was checked for all examined SNPs using chi-square test (χ^2^ test) by comparing the observed and expected genotype frequencies. The distribution of baseline characteristics between the groups was compared for qualitative variables using χ^2^ test and for quantitative variable (age) by the student’s *t*-test. Logistic regression test was applied to evaluate the association of various genotypes and alleles, combined genotypes and SNP- smoking combination with disease risk and response to imatinib. All statistical tests were performed using the SPSS software version 22 (SPSS IBM, New York, USA). LD analysis and calculation of haplotype frequencies were performed using online software SNPStats (https://www.snpstats.net) and HaploView ver. 4.2 (https://www.broadinstitute.org/ haploview/haploview). *p* values of less than 0.05 were considered as a statistically significant value.

**Table 1:** Primer sequences for the *ABCB1* SNPs analyzed in this study

**SNP**	**Primer sequence**	**Annealing temperature**	**Restriction enzyme**	**Fragment** **length (bp)**
c.3435C>T	F: 5'TGGCAAAGAAATAAAGCGAC3' R: 5'CTAACCCAAACAGGAAGTGTG3'	58 °C	MboI	CC: 141,221 TT: 362 CT: 141,221, 362
				
c.1236C>T	F: 5'TCCAGCTCTCCACAAAATATCAC3' R: 5'ATGGTCCTAATATCCTGTCCATC3'	65 °C	ECO01091	CC: 365,265 TT: 630 CT: 630,365,265

**Ethical statement**

The above-mentioned sampling protocols were approved by the Research Ethics Committee of Pasteur Institute of Iran, Tehran, Iran (ethical code: ir.pii.rec.1397.56). Written informed consents were provided by all the patients. 

## RESULTS

**Baseline features of the studied population**

The subjects under study consisted of 98 CML patients and 100 controls that 54 (55.1%) patients were in the IM non-responder group and 44 (44.9%) patients in the IM responder group. There was no significant difference in the mean age between the controls and patients (*p* = 0.89), as well as between the responders and non-responders (*p* = 0.54). However, the smoking status difference was significant between the controls and patients (*p* = 0.00) and between non-responders and responders (*p* = 0.003). The number of active and passive smokers among the patients and non-responders were higher than the controls and responders, respectively. The baseline features of the patient and control subjects are shown in Table 2.

**Allele and genotype frequencies of SNPs**

All the SNPs were in agreement with the Hardy-Weinberg equilibrium, both in the CML patients and controls (*p* > 0.05). The genotypic distribution of *ABCB1* (c.1236C>T and c.3435C>T) polymorphisms among the cases and controls and also among IM response groups are shown in Tables 3 and 4, respectively. The genotypic and allelic frequencies of *ABCB1* (c.1236C>T and c.3435C>T) polymorphisms were similar among CML patients and healthy individuals (*p* > 0.05), indicating no relationship between these SNPs and CML risk (Table 3). There was no significant difference in the genotype frequencies of *ABCB1 *c.3435C>T polymorphism between IM responders and IM non-responders groups (*p* > 0.05; Table 4). The patients with c.1236CC genotype had more than threefold risk of resistance (OR = 3.94; 95% CI: 1.08-14.37; *p *= 0.038) than CT/TT genotype. The frequency of IM resistance was correlated with the number of C alleles at locus *ABCB1* 1236. There was no significant difference in the allele frequencies of *ABCB1* SNPs between the controls and patients and also response groups (Tables 3 and 4).

**Table 2:** Baseline features of the studied subjects

**Features**	**Controls** **(n = 100)**		**Patients** **(n = 98)**	***P*** **value**	**Non-CCyR** **(n = 54)**	**CCyR** **(n = 44)**	***p*** **value**
Individuals Age (y) Mean ± SD	44.3 ± 14.96		44.57 ± 15.02	0.89	43.27 ± 16.08	45.61 ± 13.72	0.54
Sex Male n (%) Female n (%)	52 (52) 48 (48)		55 (56.1) 43 (43.9)	0.561	29 (53.7) 25 (46.3)	26 (59.1) 18 (40.9)	0.593
Smoking statues Active n (%) Passive n (%) Never n (%)	11 (11) 7 (7) 82 (82)		29 (29.6) 25 (25.5) 44 (44.9)	**0.00**	21(38.9) 17 (31.5) 16 (29.6)	8 (18.2) 8 (18.2) 28 (63.6)	**0.003**
Follow-up duration (month) Mean ± SD					56.37 ± 24.55	63.36 ± 39.8	
IM treatment duration (month) Mean ± SD					51.15 ± 22.43	59.07 ± 38.22	

Bold indicates the significant values.

**Table 3:** Analysis of the association between *ABCB1* SNPs and CML risk

	**SNP/model**	**Genotype**	**Controls ** **n = 100 (%)**	**Patients ** **n = 98 (%)**	***p*** ^*^ **value**	**OR** ^*^ **(95% CI)**
**c.1236C>T**	Codominant	TT	29 (29.0)	25 (25.5)	Ref	1
		CT	53(53.0)	54 (55.1)	0.88	1.08 (0.53-2.20)
		CC	18(18.0)	19(19.4)		0.88 (0.35-2.21)
					
	Dominant	TT	29 (29.0)	25 (25.5)	Ref	1
		CT/CC	71 (71.0)	73 (74.5)	0.93	1.03 (0.52-2.03)
					
	Recessive	TT/CC	82 (82.0)	79 (80.6)	Ref	1
		CT	18 (18.0)	19 (19.4)	0.65	0.83 (0.38-1.84)
					
	Alleles	T	111 (51.6)	104 (48.4)	Ref	1
		C	89 (49.2)	92 (50.8)	0.85	0.96 (0.62-1.476)
						
**c.3435C>T**	Codominant	TT	29 (29.0)	22 (22.4)	Ref	1
		CT	49 (49.0)	57 (58.2)	0.66	1.13 (0.54-2.33)
		CC	22 (22.0)	19 (19.4)		0.78 (0.31-1.94)
					
	Dominant	TT	29 (29.0)	22 (22.4)	vef	1
		CT/CC	71 (71.0)	76 (77.5)	0.95	1.02 (0.51-2.05)
					
	Recessive	TT/CT	78 (78.0)	79 (80.6)	Ref	1
		CC	22 (22.0)	19 (19.4)	0.4	0.72 (0.34-1.55)
					
	Alleles	T	107 (51.4)	101 (48.6)	Ref	1
		C	93 (49.5)	95 (50.5)	0.66	0.9 (0.59-104)

Ref, reference; ^*^logistic regression model adjusted for sex, age, and smoking status

**Haplotype inference**

**Fig. 1:**
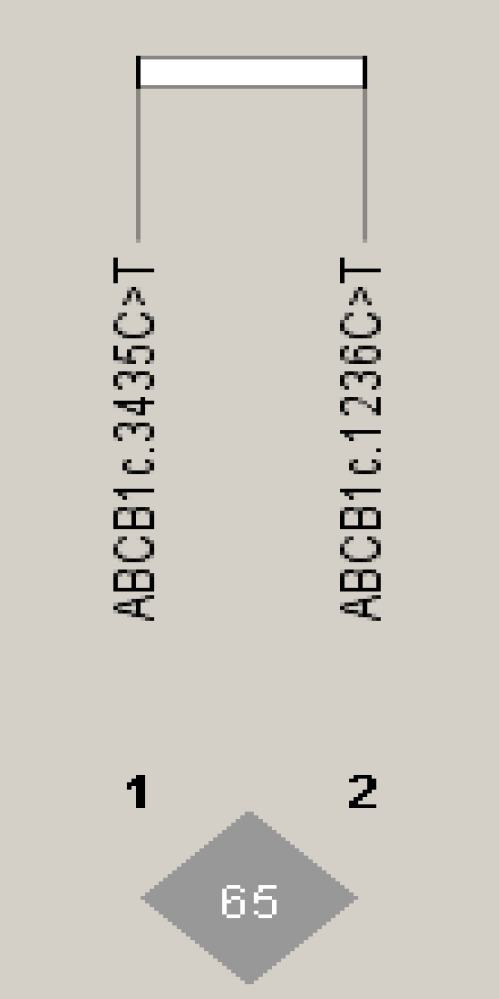
Haploview LD map. The plots show the D' values of *ABCB1* polymorphisms (c.3435C>T and c.1236C>T; D' = 0.657).

 LD between *ABCB1 *c.1236C>T and c.3435C>T polymorphisms was estimated with SNPStats online software. A moderate LD was observed between *ABCB1* polymorphisms (D' = 0.657, r^2 ^= 0.40, LOD [log odds] = 20.45; Fig. 1). The most frequent *ABCB1* haplotypes found both in the patients and controls were 1236T/3435T (44.2%) and 1236C/3435C (37.4%), respectively. None of the haplotypes were associated with CML risk and imatinib response. The haplotype frequencies of *ABCB1* polymorphisms are shown in Table 5. 

**Assessment of combined genotypes**

To evaluate the joint effect of double combinations of SNPs on CML susceptibility and CCyR to imatinib, we considered TT/CT genotype as a reference genotype, according to the recessive model for *ABCB1* SNPs. The distribution of combined genotypes in the patients and healthy controls are shown in Table 6. No statistically significant association was found between combined genotypes of SNPs and CML risk and IM resistance. There was a trend to almost significance for combination of 1236CC and 3435CC genotypes (*p* = 0.06) between non-responder and responders. This genotype combination increased more than fivefold risk of resistance.

**Gene-smoking combination**

The joint effect of *ABCB1* polymorphisms and smoking on the risk of IM resistance and CML was evaluated, and its results are shown in Table 7. The reference group was non-smoker subjects with TT/CT genotypes for the two SNPs. Also, the sum of active and passive groups was selected as smokers. The joint effect of smoking and *ABCB1 *SNPs significantly increased the risk of CML; therefore, the smoker subjects with c.1236TT/CT and c.1236CC genotypes showed higher risk for CML development (six and five folds more than reference group; OR = 6.04; 95% CI: 2.82-12.92;* p* = 0.00 and OR = 4.95; 95% CI: 1.62-15.13; *p* = 0.005, respectively). Moreover, c.3435TT/CT genotype and CC genotype among smoker individuals significantly increased CML risk (OR = 6.01; 95% CI: 2.8-12.91; *p* = 0.00 and OR = 4.36; 95% CI: 1.41-13.52;* p* = 0.011, respectively).

**Table 4:** Analysis of the association between *ABCB1* SNPs and IM response groups

	**SNP/model**	**Genotype**	**CCyR ** **n = 44 (%)**	**Non-CCyR ** **n = 54 (%)**	***p*** ^*^ **value**	**OR** ^*^ ** (95% CI)**
**c.1236C>T**	Codominant	TT	12 (27.3)	13(24.1)	Ref	1
		CT	28(63.6)	26 (48.1)	0.11	0.08 (0.29-2.22)
		CC	4(9.1)	15(27.8)		3.4 (0.79-14.6)
					
	Dominant	TT	12 (27.3)	13 (24.1)	Ref	1
		CT/CC	32 (72.7)	41 (75.9)	0.82	1.12 (0.42-2.98)
					
	Recessive	TT/CT	40 (90.9)	39 (72.2)	Ref	1
		CC	4 (9.1)	15 (27.8)	**0.038**	3.94 (1.08-14.37)
					
	Alleles	T	52 (59.1)	52 (48.1)	Ref	1
		C	36 (40.9)	56 (51.9)	0.19	1.51 (0.81-2.80)
						
**c.3435C>T**	Codominant	TT	13 (29.6)	9 (16.7)	Ref	1
		CT	26 (59.1)	31 (57.4)	0.16	1.3 (0.42-3.99)
		CC	5 (11.4)	14 (25.9)		4.02 (0.89-18.06)
					
	Dominant	TT	13 (29.6)	9 (16.7)	Ref	1
		CT/CC	31 (70.5)	45 (83.3)	0.36	1.66 (0.56-4.90)
					
	Recessive	TT/CT	39 (88.6)	40 (74.1)	Ref	1
		CC	5 (11.4)	14 (25.9)	0.062	3.32 (0.94-11.72)
					
	Alleles	T	52 (59.1)	49 (45.4)	Ref	1
		C	38 (40.9)	59 (54.6)	0.11	1.66 (0.89-3.1)

Ref: reference; ^*^logistic regression model adjusted for sex, age, and smoking status

Regarding IM response, *ABCB1 *c.1236TT/CT genotype and also CC genotype in the smoker patients increased the treatment failure risk with different values (OR = 5.36; 95% CI: 1.93-14.88;* p* = 0.001 and OR = 15.78; 95% CI: 2.82-88.43; *p* = 0.002, respectively). The joint effect of smoking with *ABCB1 *c.3435TT/CT and c.3435CC genotypes significantly elevated imatinib resistance in smoker patients (OR = 5.61; 95% CI: 1.95-16.12; *p* = 0.001 and OR = 13.58; 95% CI: 2.41-76.52; *p* = 0.002, respectively).

## DISCUSSION

Despite the supreme results of imatinib in the treatment of CML patients, the response in patients is very heterogeneous, which may be in part due to pharmacogenetic variability^[^^18^^]^. IM is a substrate of P-gp efflux pump and is encoded by *ABCB1* gene; therefore, functional alteration in this gene might illustrate variable responses to this drug^[^^19^^]^. SNPs in *ABCB1* gene could change protein function and influence the performance of sorption or elimination^[^^20^^]^. Therefore, in this study, we explained the usefulness of SNPs and their combination with smoke as a synergistic factor to identify CML risk, especially the risk of resistance.

In our studied population, no significant association was observed between different genotypes (*ABCB1 *1236CT and *ABCB1 *3435CT) and the risk of CML development. As far as we know, the role of *ABCB1* polymorphisms in the development of CML has not yet been investigated in earlier studies. Only the study by Abuhaliema *et al.*^[^^11^^]^ regarding the association of mentioned polymorphisms with cancer risk on Jordanian women showed that the genetic polymorphism *ABCB1* 3435CT, but not 1236CT, was associated with the increased risk of breast cancer. 

**Table 5:** Association of haplotypes with CML risk and IM response

**c.1236** **C>T**	**c.3435** **C>T**	**Total freq.**	**Control** **Freq.**	**Case freq.**	***p*** **value**	OR* (95%CI)	**CCyR freq.**	**Non-CCyR freq.**	**Total freq.**	***p*** **value**	OR* (95%CI)
T	T	0.4428	0.4691	0.4691	Ref.	1	0.5002	0.3441	0.4144	-	1
C	C	0.3746	0.3791	0.3791	0.66	0.89 (0.54-1.48)	0.3184	0.4089	0.3684	0.07	2.11 (0.94-4.72)
T	C	0.1001	0.0859	0.0859	0.86	1.08 (0.47-2.49)	0.0907	0.1374	0.1162	0.2	2.36 (0.63-8.81)
C	T	0.0825	0.0659	0.0659	0.58	1.29 (0.53-3.15)	0.0907	0.1096	0.1009	0.41	1.72 (0.48-6.24)

freq, frequency; Ref: reference; ^*^logistic regression model adjusted for sex, age, and smoking status

**Table 6:** Analysis of the association between combined genotypes, CML risk, and IM response

**c.1236** **C>T**	**c.3435** **C>T**	**Control ** **n= 100 (%)**	**Patient** **n = 98 (%)**	***p*** ** value**	OR* **(95% CI)**	**CCyR ** ** n = 44 (%)**	**Non-CCyR** ** n = 54 (%)**	***p*** **value**	OR* ** (95% CI)**
TT/CT	TT/CT	72 (72.0)	70 (71.4)	Ref	1	37 (84.1)	33 (61.1)	Ref	1
TT/CT	TT/CT	10 (10.0)	9 (9.2)	0.64	0.78 (0.27-2.23)	3 (6.8)	6 (11.1)	0.25	2.69 (0.50-14.31)
CC	CC	6 (6.0)	9 (9.2)	0.94	1.05 (0.31-3.50)	2 (4.5)	7 (13.0)	0.16	3.53 (0.60-20.61)
CC	CC	12 (12.0)	10 (10.2)	0.45	0.68 (0.25-1.85)	2 (4.5)	8 (14.8)	0.06	5.59 (0.93-33.55)

Ref, reference; ^*^logistic regression model adjusted for sex, age, and smoking status

**Table 7:** SNP-smoking combination and CML risk, IM response

	**SNP/** **gen.**	**Smoking** ** status**	**Control** **n = 100 (%)**	**Patient** **n = 98 (%)**	***p*** **value**	OR* **(95% CI)**	**CCyR** **n = 44 (%)**	**Non-CCyR ** **n = 54** **(%)**	***p*** ** value**	OR* **(95% CI)**
**c.1236C>T**	TT/CT	N/S S	69 (69) 14 (14)	38 (38.78) 41 (41.84)	Ref **0**	**1** 6.04 (2.82-12.92)	26 (59.1) 14 (31.8)	12 (22.22) 27 (50)	Ref **0.001**	**1** 5.36 (1.93-14.88)
									
	CC	N/S S	12 (12) 5 (5**)**	6 (6.12) 13 (13.26)	0.75 **0.005**	0.84 (0.30-2.41) 4.95 (1.62-15.13)	2(4.54) 2(4.54)	4 (7.4) 11 (20.38)	0.078 **0.002**	5.45 (0.83-35.94) 15.78 (2.82-88.43)
										
**c.3435C>T**	TT/CT	N/S S	65 (65) 13 (13)	37 (37.75) 42 (42.86)	Ref **0**	**1** 6.01 (2.8-12.91)	25 (56.82) 14 (31.82)	12 (22.22) 28 (51.86)	Ref **0.001**	**1** 5.61 (1.95-16.12)
									
	CC	N/S S	17 (17) 5 (5)	7 (7.14) 12 (12.25)	0.51 **0.011**	0.72 (0.27-1.91) 4.36 (1.41-13.52)	3 (6.82) 2 (4.54)	4 (7.4) 10 (18.52)	0.122 **0.002**	4.03 (0.69-23.61) 13.58 (2.41-76.52)

Gen, genotype; N/S, non-smoker; S, smoker; Ref, reference; ^*^logistic regression model adjusted for sex and age

In the current study, cytogenetic resistance was significantly related to the patients who had 1236CC genotype. In other words, this genotype increased almost fourfold risk of IM resistance in the non-responder group, whereas no correlation was observed between 3435CT and IM response. In agreement with our results, a meta-analysis suggested that *ABCB1 *c.1236CT polymorphism is a risk factor for non-optimal clinical response in Asian CML patients^[^^21^^]^. Our results is also in accordance with the findings of Au *et al.*^[^^5^^]^, Dulucq *et al.*^[^^18^^]^, and Deenik *et al.*^[^^22^^] ^who reported that the patients with 1236CC genotype were associated with IM resistance, and those with 1236TT genotype showed higher probability to achieve IM response. In contrast, in several studies, no association was found between 1236CT and 3435CT genotypes and IM response^[^^13^^,^^23^^,^^24^^]^. 

Regarding the c.3435 CC genotype, Lardo *et al.*^[^^25^^]^ found the association of this genotype with the complete molecular response, while Ni *et al.*^[^^26^^]^ indicated the association of c.3435TT with the increased risk of IM resistance. In our study, no association was detected between double combinations of genotypes with CML risk and IM response, except for the hybrid pattern of 1236CC/3435CC, which seems to be related with the higher risk of IM resistance. In former studies, the effect of genotype combinations on IM response and CML risk have not been studied; nonetheless, it seems that genotype combinations and their interaction are effective predictors for response to imatinib. Our results showed that in both SNPs, 1236C>T and 3435C>T, smoke has a significant synergistic role in all the genotypes and leads to the increased risk of CML and IM resistance (5-15fold) among non-responders, compared to responders. It is thought that cigarette smoke can alter the expression and/or the activity of transporter genes such as *ABCB1* gene^[^^27^^]^. 

In conclusion, this is the first study on CML patients that reveals the influence of an environmental factor, like the cigarette smoke, on the *ABCB1* transporter gene, which may leads to the enhancement of CML risk and IM resistance. Our results also suggest that 1236CC genotype has clinical importance in the prediction of treatment outcome with IM, and smoke could have an additive role in the CML risk and IM resistance.
